# The role of miR-100 in regulating apoptosis of breast cancer cells

**DOI:** 10.1038/srep11650

**Published:** 2015-07-01

**Authors:** Yi Gong, Tianliang He, Lu Yang, Geng Yang, Yulei Chen, Xiaobo Zhang

**Affiliations:** 1 Key Laboratory of Animal Virology of Ministry of Agriculture and College of Life Sciences, Zhejiang University, Hangzhou 310058, The People’s Republic of China

## Abstract

Breast cancer is a serious health problem worldwide. Inhibition of apoptosis plays a major role in breast cancer tumorigenesis. MicroRNAs (miRNAs) play crucial roles in the regulation of apoptosis. However, the regulation of breast cancer apoptosis by miRNAs has not been intensively investigated. To address this issue, the effect of miR-100 on the cell proliferation of different breast cancer cells was characterized in the present study. The results showed that miR-100 was significantly upregulated in SK-BR-3 cells compared with other human breast cancer cells (MCF7, MDA-MB-453, T47D, HCC1954 and SUM149). Silencing miR-100 expression with anti-miRNA-100 oligonucleotide (AMO-miR-100) initiated apoptosis of SK-BR-3 cells *in vitro* and *in vivo*. However, the overexpression of miR-100 led to the proliferation inhibition of the miR-100-downregulated breast cancer cells. Antagonism of miR-100 in SK-BR-3 cells increased the expression of *MTMR3*, a target gene of miR-100, which resulted in the activation of *p27* and eventually led to G2/M cell-cycle arrest and apoptosis. The downregulation of miR-100 sensitized SK-BR-3 cells to chemotherapy. Therefore, our finding highlights a novel aspect of the miR-100-*MTMR3*-*p27* pathway in the molecular etiology of breast cancer.

Breast cancer poses a serious health problem, with mortality attributed to the metastatic spread of the cancer to vital organs, such as the lung, liver and bone^1^,^2^ . Breast cancer is one of the most common malignant cancers, and the most common among women^3^,^4^, with around one million new cases each year. In addition to several types of surgical therapies, the current treatment for patients with breast cancer requires judiciously applied serial endocrine, chemotherapeutic and biological therapies to produce some efficacy and a reduced death rate^5^. Surgery is the primary treatment for patients with early breast cancer and has improved patient long-term survival, but it is ineffective for individuals with advanced disease^6^. Many non-surgical treatments for breast cancer have been investigated, however, traditional non-surgical therapies are associated with significant toxicity^5^. Therefore, the development of novel treatments is required.

Tumorigenesis is the result of uncontrollable cell proliferation, which can be caused by various carcinogenic factors. The inhibition of apoptosis significantly promotes tumorigenesis^7^,^8^. Tumors are virtually a kind of genetic disease, as the activation of oncogenes and inactivation of tumor suppressor genes, combined with the mutation of apoptosis regulation and DNA repair genes, are thought to be the cause of tumorigenesis^9^,^10^. The discovery of non-coding small RNAs led to many studies suggesting that they have important roles in the regulation of many diseases, including tumours^11^. MicroRNAs (miRNAs), typically 19–25 nucleotides in length, are a class of small non-coding RNAs that can downregulate the expression of specific target genes^12^,^13^,^14^. The fact that around 50% of miRNA genes are located in tumour-associated genomic regions suggests that miRNAs have a significant function in tumourigenesis^14^,^15^. Computational predictions of miRNA target genes reveal that approximately one third of all human protein-encoding genes may be regulated by miRNAs, including a wide range of genes involved in tumourigenesis^16^. Recently, researches have revealed that virtually all examined tumour types have abnormal miRNA expression, indicating that miRNAs may be involved in the regulation of some biological functions in cancer cells. Since avoiding apoptosis is a critical property of malignant tumours and miRNAs are well known to have key roles in apoptosis regulation^17^,^18^, it is likely that miRNAs promote tumour formation by regulating apoptosis and this needs to be addressed. Given that most chemotherapeutic drugs kill cancer cells through apoptosis and that miRNAs are involved in the regulation of apoptosis, it is likely that miRNAs are an effective target for cancer therapies.

Despite the biological function of miRNAs becoming increasingly apparent, the role of miRNAs in regulating apoptosis of cancer cells, such as breast cancer cells, has not been intensively investigated. To address this issue, the regulation of apoptosis mediated by miR-100, a miRNA associated with apoptosis regulation^19^, was investigated in this study. The results showed that miR-100 was significantly upregulated in SK-BR-3 cells, when compared with five other human breast cancer cells. It was further revealed that the role of miR-100 in regulating apoptosis was different in various breast cancer cells.

## Results

### The involvement of miR-100 in the regulation of apoptosis in breast cancer cells

**Figure 1:**
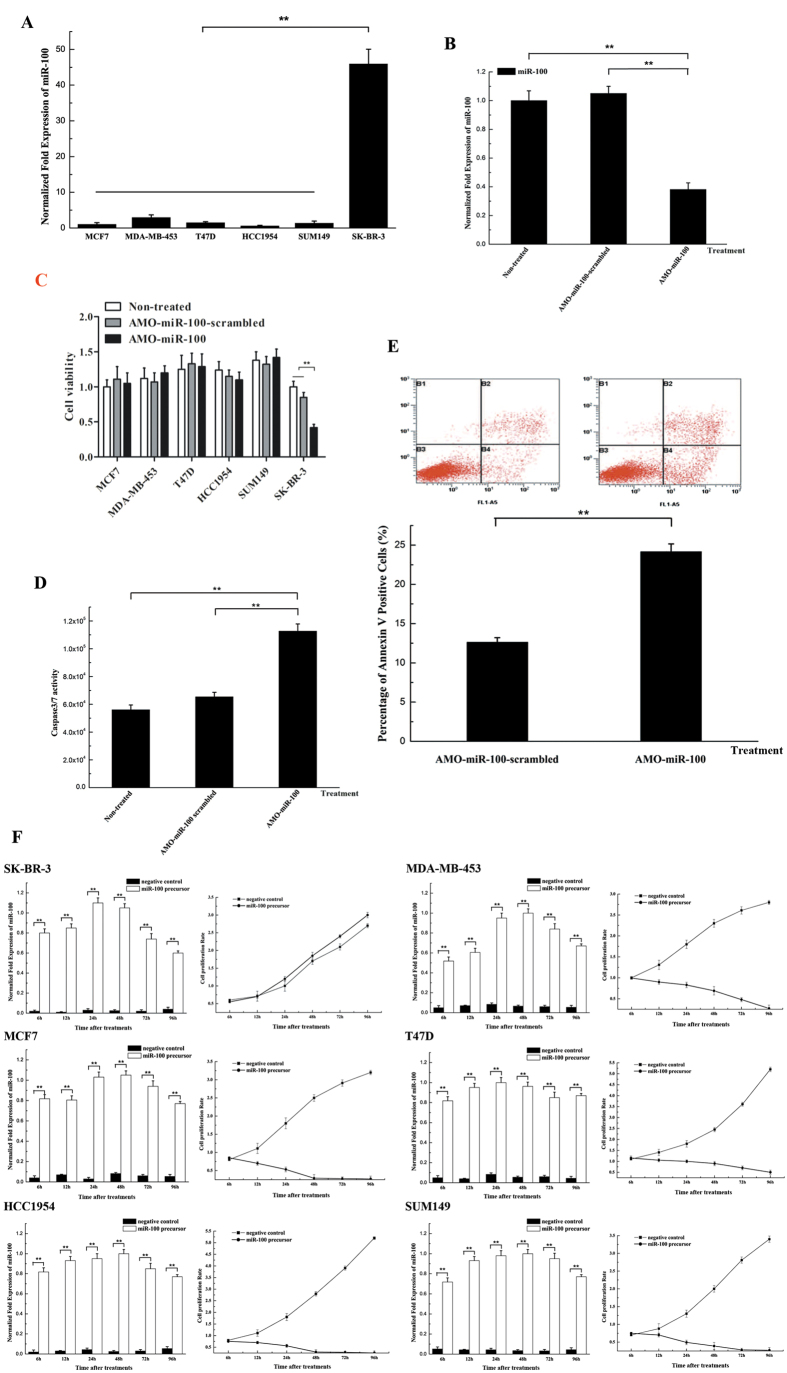
The role of miR-100 in the regulation of apoptosis in breast cancer cells. (**A**) The expression of miR-100 in breast cancer cells. The expression level of miR-100 in different cell lines including SK-BR-3, T47D, SUM149, HCC1954, MDA-MB-453 and MCF7 was examined with quantitative real-time PCR. (**B**) Silencing of miR-100 expression in breast cancer cells. AMO-miR-100 or AMO-miR-100-scrambled was transfected into SK-BR-3 cells, followed by detection of miR-100 expression using real-time PCR at 48 h after transfection. Non-treated cells were used as controls. (**C**) The effect of miR-100 silencing on cell viability. Cell viability was evaluated at 48 h after transfection of breast cancer cells with the AMOs. (**D**) Influence of miR-100 silencing on caspase 3/7 activity in breast cancer cells. The SK-BR-3 cells at 36 h after transfection of AMO-miR-100 or AMO-miR-100-scrambled were subjected to the Caspase-Glo 3/7 assay to evaluate apoptosis. (**E**) The effects of miR-100 downregulation on apoptosis using Annexin V assays. Apoptosis was examined by flow cytometry at 48 h after transfection of AMOs. (**F**) Overexpression of miR-100 in breast cancer cells. SK-BR-3, MCF7, HCC1954, MDA-MB-453, T47D and SUM149 cells were transfected with the miR-100 precursor or a negative control. At different times after transfection, the cells were subjected to real-time PCR to detect miR-100 and the effects of miR-100 overexpression on cell proliferation were analysed. In all panels, plotted data referred to the means ± standard deviations of triplicate assays and asterisks represented statistically significant differences (**p < 0.01).

To explore the role of miR-100 in regulating apoptosis of breast cancer, the expression levels of miR-100 in different breast cancer cell lines were examined, including MCF7, MDA-MB-453, T47D, HCC1954, SUM149 and SK-BR-3. The results showed that miR-100 was significantly upregulated in SK-BR-3 cells and downregulated in MCF7, MDA-MB-453, T47D, HCC1954 and SUM149 cells (Fig. 1A), suggesting that the miR-100-mediated apoptotic pathway might be different in various cancer cells. To knock down the expression of miR-100, the breast cancer cells were transfected with anti-miRNA-100 oligonucleotide (AMO-miR-100), respectively. It was found that miR-100 expression was specifically reduced by AMO-miR-100 (Fig. 1B). Silencing miR-100 led to a significant decrease in cell viability and a significant increase in caspase 3/7 activity in SK-BR-3 cells compared with controls (Non-treated and AMO-miR-100-scrambled) (Fig. 1C,D), indicating that miR-100 was involved in inhibiting apoptosis of breast cancer cells. Annexin V assays revealed that suppressing miR-100 increased the proportion of cells in early apoptosis of SK-BR-3 cells, when compared with the control (AMO-miR-100-scrambled) (Fig. 1E). However, the miR-100 silencing had no effect on the cell viability of MCF7, MDA-MB-453, T47D, HCC1954 and SUM149 cells, in which miR-100 was downregulated. These findings indicated that miR-100 had an important role in the regulation of apoptosis of SK-BR-3 cells.

To evaluate the effects of miR-100 overexpression on breast cancer cells, the miR-100 precursor was transfected into breast cancer cells. The expression of miR-100 was significantly increased in miR-100 precursor transfected cells relative to cells transfected with the miR-100 negative control (Fig. 1F). Interestingly, the overexpression of miR-100 had a slight stimulatory effect on cell growth in SK-BR-3 cells, while seriously damaging other breast cancer cells (Fig. 1F).

These data indicated that miR-100 was specifically upregulated in SK-BR-3 cells and its function in regulating apoptosis was different in various breast cancer cells.

### The effects of miR-100 on the tumourigenesis of breast cancer *in vivo*

**Figure 2:**
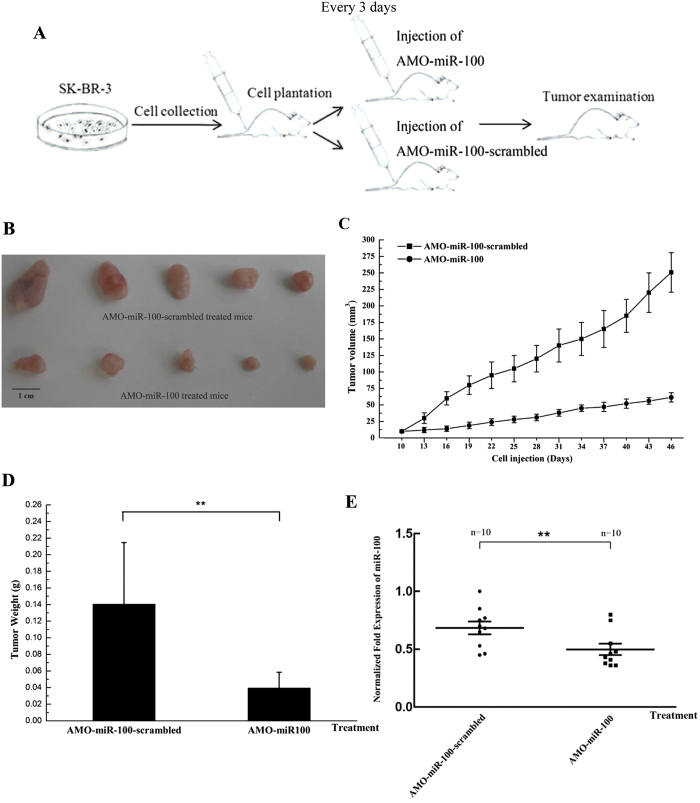
The effects of miR-100 on the tumourigenesis of breast cancer cells *in vivo.* (**A**) A flow diagram of the *in vivo* experiments. (**B**) The effects of the inhibition of miR-100 expression on solid tumours in nude mice. SK-BR-3 cells were injected into nude mice and 10 d later AMO-miR-100-scrambled or AMO-miR-100 was subcutaneously and intravenously injected into mice. The tumour images were obtained 46 d after cell injection. The effect of miR-100 silencing on tumour volume (**C**) and weight (**D**). The mice were treated with AMOs and then the tumour volume and weight was examined 46 d after cell injection. The data were the means results from 10 mice. (**E**) The expression of miR-100 in tumour tissues. Solid tumours treated with AMOs were subjected to real-time PCR to evaluate the miR-100 expression level. The data represented the means ± standard deviations of triplicate assays. The statistically significant differences between different treatments were indicated with asterisks (**p < 0.01).

In the miR-100-downregulated breast cancer cells, the previous study has revealed that miR-100 can suppress tumuorigenesis *in vivo*^20^. In this study, to evaluate the effects of miR-100-mediated apoptosis regulation on breast cancer tumourigenesis *in vivo*, SK-BR-3 cells were injected into nude mice, which were further treated with AMO-miR-100 or AMO-miR-100-scrambled and tumour development was monitored (Fig. 2A). Tumour development was significantly reduced in mice treated with AMO-miR-100, compared with mice treated with the control (AMO-miR-100-scrambled) (Fig. 2B). This indicated that miR-100 antagonism could inhibit the development of breast cancer *in vivo*. In addition, the volume and weight of tumours was significantly lower in mice treated with AMO-miR-100 (Fig. 2C,D). These results suggested that miR-100 was involved in tumour development *in vivo*.

To examine the expression of miR-100 in mice treated with AMO-miR-100 or AMO-miR-100-scrambled, the total RNAs from the solid tumours were extracted and real-time PCR was performed. The results revealed that the expression of miR-100 was significantly reduced by AMO-miR-100 (Fig. 2E), which suggested that the reduced tumour development resulted from miR-100 silencing. Therefore, miR-100 antagonism could inhibit tumourigenesis of SK-BR-3 cells *in vivo*. The effects of miR-100 on tumourigenesis were inconsistent in different breast cancer cells.

### The apoptotic target of miR-100 in breast cancer cells

**Figure 3:**
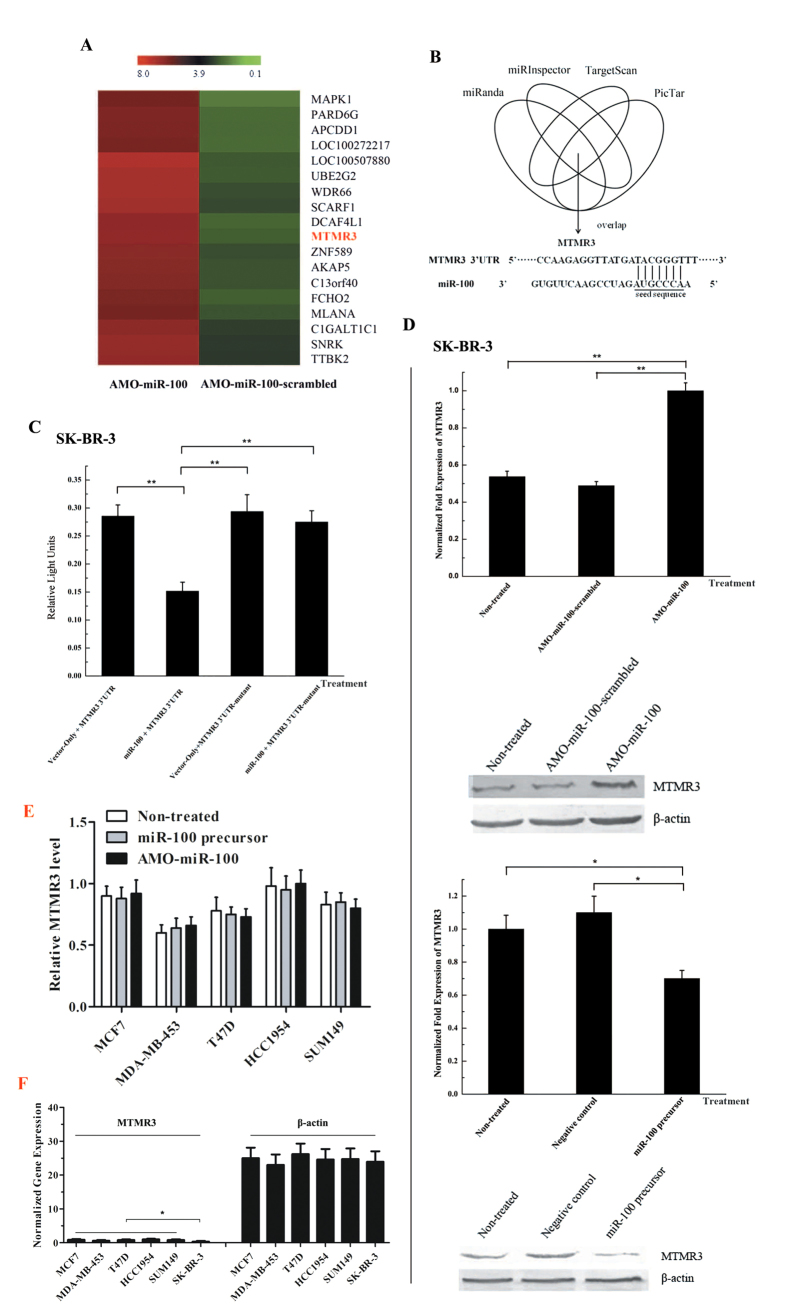
The target gene of miR-100. (**A**) Screening of miR-100 target genes with DNA microarray analyses. The SK-BR-3 cells were treated with AMO-miR-100 or AMO-miR-100-scrambled. Forty-eight hours later, total RNA was extracted from the cells and was analysed by DNA microarray. Examples of the normalized hybridization signals of potential target genes were presented. Signal value was indicated at the top of every lane. Lane headings showed the treatments. (**B**) The target gene prediction of miR-100 based on Targetscan, miRanda and Pictar algorithms. (**C**) The direct interaction between miR-100 and *MTMR3* 3′UTR. The SK-BR-3 cells were co-transfected with miR-100 and a luciferase reporter fused with MTMR3 3′UTR and firefly and renilla luciferase activities were analysed. Vector only and MTMR3 3′UTR-mutant were included as controls in the co-transfections. (**D**) The effects of miR-100 silencing or overexpression on *MTMR3* gene expression in SK-BR-3 cells. After transfection of miR-100 precursor or negative control AMOs, the levels of *MTMR3* gene expression were detected by quantitative real-time PCR and western blot. (**E**) The effects of miR-100 silencing or overexpression on *MTMR3* expression in MCF7, MDA-MB-453, T47D, HCC1954 and SUM149 cells. The expression level of *MTMR3* in different cell lines was examined with quantitative real-time PCR after treated with AMO-miR-100 or miR-100 precursor. (**F**) The expression of *MTMR3* in breast cancer cells. The expression level of *MTMR3* was examined with quantitative real-time PCR. β-actin was used as a control. In all panels, the data represented the means ± standard deviations of triplicate assays and asterisks indicated statistically significant differences (*p < 0.05; **p < 0.01).

To explore the miR-100-mediated apoptotic-signalling pathway in breast cancer cells, the target genes of miR-100 were screened using a DNA microarray. The results suggested that *MTMR3* (myotubularin related protein 3) might be a target gene of miR-100 (Fig. 3A). In addition, the target gene prediction indicated that *MTMR3* was a target of miR-100 (Fig. 3B). A dual luciferase reporter assay, in which the wild-type *MTMR3* 3’UTR was expressed with luciferase, revealed that miR-100 could significantly decrease the expression of luciferase. However, no reduction was seen when luciferase was expressed with a *MTMR3* 3’UTR mutant (Fig. 3C). This indicated the existence of a direct interaction between miR-100 and *MTMR3* mRNA.

The interaction between miR-100 and *MTMR3* mRNA was further evaluated in breast cancer cells *in vivo*. It was revealed that miR-100 knockdown resulted in a significant upregulation of *MTMR3* mRNA and its encoded protein in SK-BR-3 cells (Fig. 3D). Conversely, miR-100 overexpression led to a significant decrease of *MTMR3* expression in SK-BR-3 cells (Fig. 3D). However, the silencing or overexpression of miR-100 took no effect on the *MTMR3* expression in the miR-100-downregulated breast cancer cells (MCF7, MDA-MB-453, T47D, HCC1954 and SUM149) (Fig. 3E). We detected the expression level of *MTMR3* in the breast cancer cells and found significantly decreased levels in SK-BR-3 cells compared with other breast cancer cells (Fig. 3F). The data were consistent with the expression profiles in breast cancer cells (Fig. 1A), suggesting that *MTMR3* was a target gene of miR-100 in SK-BR-3 cells. As previously reported^20^, miR-100 could target the *SMARCA5* gene in MCF7 and HMLE cells, in which miR-100 was downregulated. In this context, miR-100 targeted different genes in various breast cancer cells. In SK-BR-3 cells, miR-100 inhibited apoptosis by negatively regulating the expression of *MTMR3*.

### The miR-100-mediated signalling pathway for apoptosis of breast cancer

**Figure 4:**
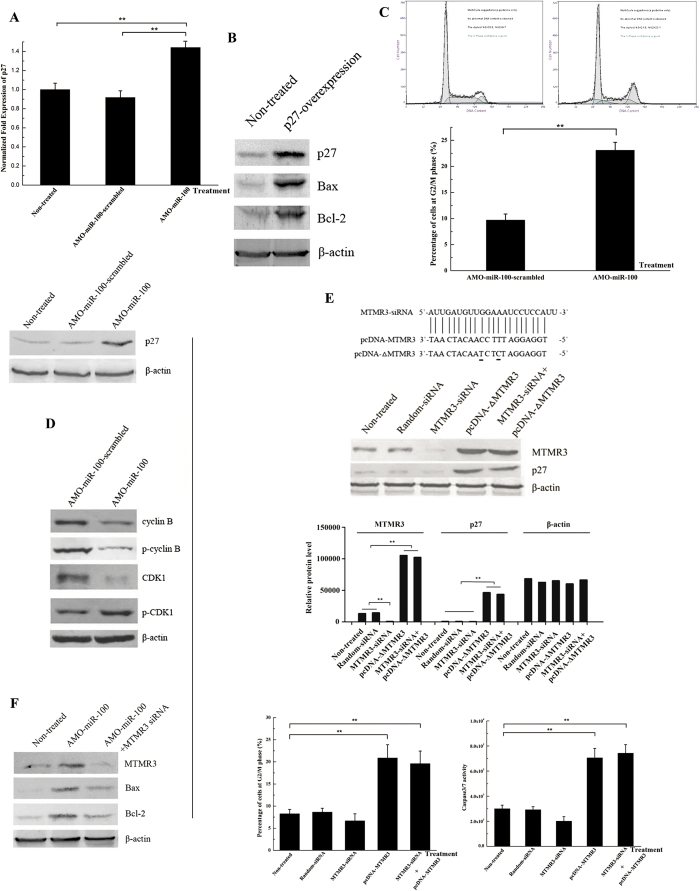
The miR-100-mediated signalling pathway for apoptosis of breast cancer cells. (**A**) The influence of miR-100 on p27 expression. SK-BR-3 cells were transfected with AMO-miR-100 or AMO-miR-100-scrambled and at 48 h after transfection the cells were subjected to real-time PCR (left) and western blot (right). (**B**) The role of p27 in regulating apoptosis of breast cancer cells. SK-BR-3 cells were transfected with pcDNA-p27 to overexpress p27 and at 36 h after transfection the cells were subjected to western blot analysis. The antibodies were indicated on the right. (**C**) The effects of miR-100 on the cell cycle of breast cancer cells. SK-BR-3 cells were transfected with AMOs and stained 48 h later with PI. The cell cycle was analysed by flow cytometry. (**D**) Detections of key effector proteins involved in the cell cycle. SK-BR-3 cells were transfected with AMO-miR-100 or AMO-miR-100-scrambled. Forty-eight hours later the proteins were analysed by western blot. β-actin was used as a control. The antibodies used were indicated on the right. (**E**) The roles of MTMR3 in the cell cycle regulation and apoptosis in breast cancer cells. The expression of MTMR3 was silenced, overexpressed and rescued in SK-BR-3 cells. The mutated nucleotides were underlined. The expression of p27 was evaluated by western blot. The proportion of cells in cell cycle arrest at the G2/M phase was analysed and caspase 3/7 activity was determined. Lane headings showed the treatments used. Non-treated cells were used as a control. (**F**) The effect of MTMR3 on breast cancer cell apoptosis induced by miR-100 inhibition. SK-BR-3 cells were transfected with AMO-miR-100 and MTMR3-siRNA and the expression of MTMR3 and the apoptotic proteins Bax and Bcl-2 were detected by western blot. (**G**) The expression level of p27 in breast cancer tissues and normal tissues. In all panels, plotted data points referred to the means ± standard deviations of triplicate assays and asterisks represented statistically significant differences (*p < 0.05; **p < 0.01).

It is reported that MTMR3 functions as a positive regulator of p27^21^, a key protein in cell cycle regulation and apoptosis^22^. To evaluate the effect of miR-100 silencing on *p27* expression, SK-BR-3 cells were treated with AMO-miR-100 and *p27* expression was analyzed. Knockdown of miR-100 resulted in significant increases of *p27* mRNA and the encoded protein when compared with non-treated cells (Fig. 4A). This indicated that miR-100 negatively regulated *p27* expression by targeting *MTMR3*, which suggested that the interaction between miR-100 and *MTMR3* was associated with cell cycle regulation and apoptosis.

To evaluate the role of p27 in the apoptosis of breast cancer cells, the *p27* gene was overexpressed in SK-BR-3 cells and apoptotic proteins were analyzed. The overexpression of p27 led to the accumulation of the pro-apoptotic proteins Bax and Bcl-2 (Fig. 4B). The data indicated that p27 played an essential role in regulating apoptosis of breast cancer cells.

To reveal the role of miR-100 in the cell cycle, the cell cycle of AMO-miR-100-treated SK-BR-3 cells was analysed. The percentage of cells in the G2/M phase following treatment with AMO-miR-100 was significantly higher than cells treated with AMO-miR-100-scrambled (Fig. 4C). This indicated that miR-100 knockdown resulted in cell cycle arrest. To further evaluate the involvement of miR-100 in the cell cycle, effector proteins of the cell cycle were analysed in miR-100-silenced cells. Both active cyclin B and inactive phospho-cyclin B were decreased when the expression of miR-100 was knocked down (Fig. 4D). In addition, it was found that active cyclin-dependent kinase 1 (CDK1) was downregulated, while the inactive phospho-CDK1 was upregulated following treatment (Fig. 4D). Therefore, miR-100 silencing inhibited the expression of active cyclin B and active CDK1, leading to the cell cycle arrest at the G2/M phase in SK-BR-3 cells.

To characterize the roles of MTMR3 in cell cycle regulation and apoptosis, the expression of the *MTMR3* gene was silenced, overexpressed and rescued in SK-BR-3 cells and the cell cycle and indicators of apoptosis were analysed. Western blots analysis indicated that *MTMR3* gene expression was knocked down by MTMR3-siRNA, while the control siRNA (random-siRNA) had no effect on *MTMR3* expression (Fig. 4E). In addition, MTMR3-siRNA reduced p27 expression (Fig. 4E). The cell cycle arrest and apoptosis in MTMR3-siRNA-treated cells showed a slight effect compared to the controls (Fig. 4E), indicating that MTMR3 effected cell cycle arrest and apoptosis. It was revealed that the expression levels of MTMR3 and p27 were upregulated when MTMR3 was overexpressed in SK-BR-3 cells, resulting in cell cycle arrest at the G2/M phase and apoptosis (Fig. 4E). To exclude the effect of endogenous MTMR3, the expression of the MTMR3 gene was inhibited by MTMR3-siRNA and the expression was then rescued by transfecting the cells with plasmid containing the *MTMR3* gene (Fig. 4E). The results showed that caspase 3/7 activity and the percentage of cells in the G2/M phase were significantly increased in transfected cells compared with the non-transfected controls (Fig. 4E). This indicated that MTMR3 had functions in cell cycle arrest and apoptosis induction in breast cancer cells.

To further confirm the role of the miR-100-*MTMR3* interaction in mediating apoptosis of SK-BR-3 cells, we transfected cells with AMO-miR-100 and a *MTMR3* specific siRNA. The results showed that MTMR3-siRNA could reduce the apoptosis caused by inhibiting miR-100, as indicated by a reduction of the pro-apoptotic proteins Bax and Bcl-2 (Fig. 4F), suggesting that targeting of *MTMR3* by miR-100 directly contributed to SK-BR-3 cell proliferation.

**Figure 5:**
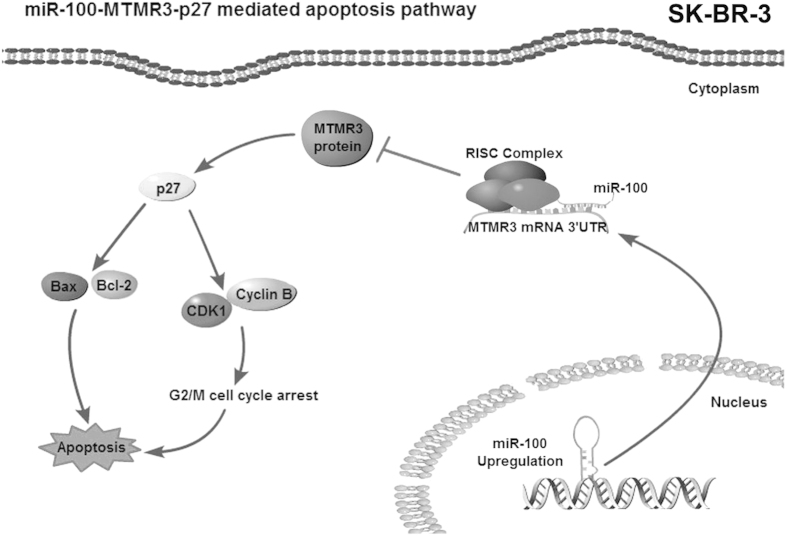
A model demonstrating how miR-100 regulates cell cycle arrest at the G2/M phase and apoptosis in SK-BR-3 cells.

Taken together, the findings demonstrated that miR-100 significantly inhibited the MTMR3-p27 signalling pathway and thereby prevented cell cycle arrest and apoptosis of SK-BR-3 cells (Fig. 5).

### The effects of miR-100 downregulation on chemotherapy of breast cancer

**Figure 6:**
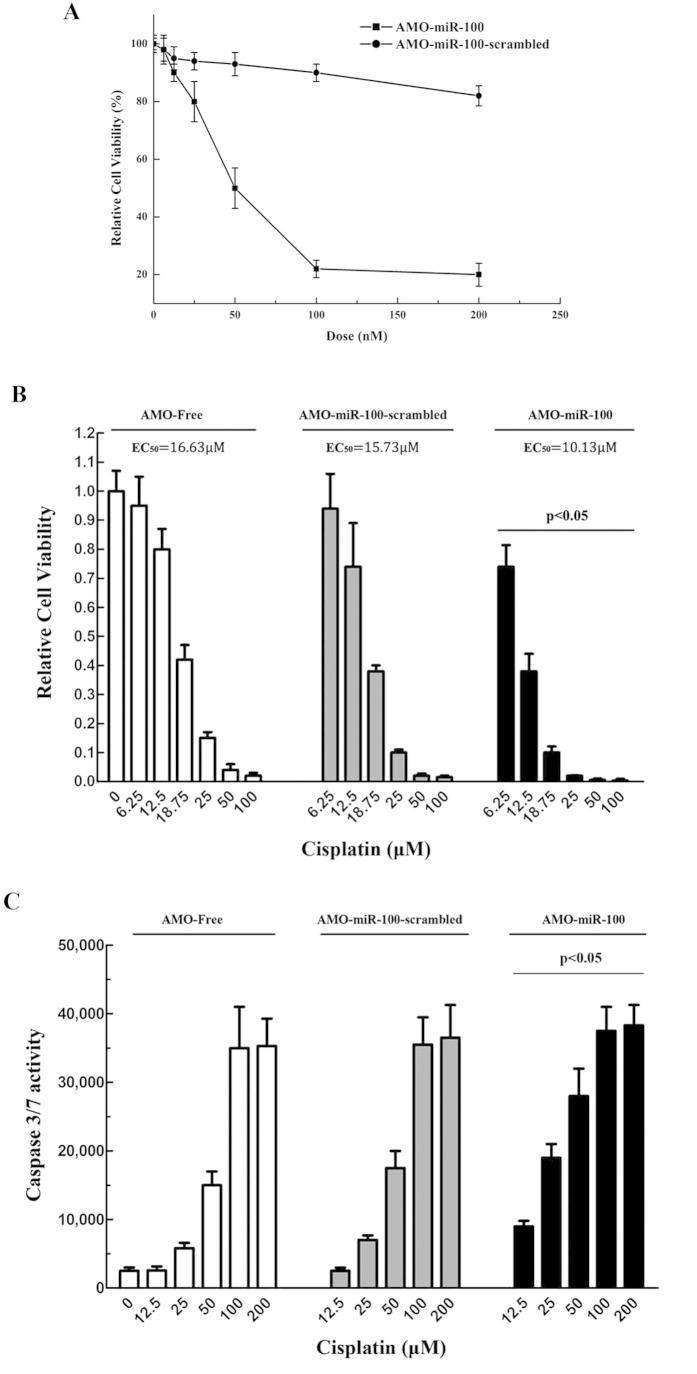
The effects of miR-100 silencing on breast cancer chemotherapy. (**A**) Relative cell viability of breast cancer cells after AMO treatments. SK-BR-3 cells were treated with AMO-miR-100 or AMO-miR-100-scrambled at different concentrations. Forty-eight h later, the cell viability was detected. (**B**) Effects of miR-100 silencing on breast cancer chemotherapy. SK-BR-3 cells were simultaneously treated with AMO and Cisplatin at different concentrations. Subsequently, the cell viability was evaluated. (**C**) Caspase 3/7 activity analysis from breast cancer cells. SK-BR-3 cells were treated with AMO and Cisplatin. Forty-eight h later the caspase 3/7 activity of cells was analysed. The statistically significant differences between different treatments were indicated with asterisks (*p < 0.05).

To evaluate the effects of miR-100 silencing as a treatment for breast cancer, SK-BR-3 cells were treated with AMO-miR-100, followed by analysis of cell viability. The results showed that the growth of SK-BR-3 cells was significantly inhibited by AMO-miR-100 treatment, compared with the control AMO-miR-100-scrambled, and that the inhibition was dose-dependent (Fig. 6A). To reveal the effects of miR-100 silencing on the efficacy of chemotherapy, SK-BR-3 cells were simultaneously treated with AMO-miR-100 and Cisplatin, a drug widely used for cancer chemotherapy, which can induce apoptosis by causing DNA damage. It was found that the EC_50_ of Cisplatin in AMO-miR-100-treated SK-BR-3 cells was significantly decreased with the combination treatment when compared with the controls (AMO-miR-100-scrambled and AMO-free) (Fig. 6B). This indicated that the downregulation of miR-100 sensitized breast cancer cells to chemotherapy. SK-BR-3 cells treated with AMO-miR-100 and Cisplatin had a significant increase in caspase 3/7 activity compared with controls (Fig. 6C). This suggested that miR-100 silencing could promote Cisplatin-induced apoptosis.

## Discussion

Abnormal expressions of miRNAs has been identified in human cancers^23^. As reported, the aberrantly expressed miRNAs can determine the initiation and progression of cancer by regulating cell proliferation, apoptosis and invasion^24^,^25^. However, the role of miRNA in the regulation of apoptosis of breast cancer is poorly understood. In this study, miR-100, a highly conserved miRNA in animals^26^, was found to be involved in breast cancer tumourigenesis that functioned by inhibiting the apoptotic activity of SK-BR-3 cells. However, miR-100 has the opposite role in regulating other types of breast cancer cells^27^,^28^. In SK-BR-3 cells, miR-100 was upregulated, but was downregulated in MCF7, T47D, HCC1954, SUM149 and MDA-MB-453 cells. Our study and the previous studies showed that the miR-100 expression level was different in various breast cancer cells^20^,^27^,^28^. In this investigation, however, the miR-100 expression level in SK-BR-3 cells was different from the previous data^27^. This discrepancy might result from the different experimental methods used. In our study, U6 was used as an endogenous standard to normalize the miR-100 expression level in breast cancer cells, while RNU24 was used to normalize the quantitative real-time PCR data in the previous study^27^. U6 is widely used for the normalization of miRNA expression level because of its constant expression level in cells in response to any treatment. The present investigation showed that the overexpression of miR-100 had a slight stimulatory effect on cell growth in SK-BR-3 cells, but seriously damaged other breast cancer cells. Our findings in SK-BR-3 cells were different from the previous results^27^, which might result from different experiment strategies. In our study, time course and the negative control of miR-100 were assayed and the overexpression of miR-100 in cells was confirmed before further investigation. The corresponding information was not described in the previous study^27^. In this study, the results indicated that silencing miR-100 triggered apoptosis in SK-BR-3 cells, leading to the suppression of tumour cell growth *in vitro* and *in vivo*. The results of our study and the previous studies revealed that miR-100 was involved in the apoptosis regulation of breast cancer cells, although the expression level of miR-100 was inconsistent in various breast cancer cells^20^,^27^,^28^. The differential expression of miR-100 suggested that the role of miR-100 in regulating apoptosis might be different in various breast cancer cells.

This study revealed that the antagonism of miR-100 mediated the apoptosis of SK-BR-3 cells by triggering the *MTMR3*-*p27* pathway, as miR-100 suppressed the *MTMR3* gene. As reported, miR-100 could target the *SMARCA5* gene in MCF7 and HMLE cells^20^, suggesting that the apoptotic pathway mediated by miR-100 was different in various breast cancer cells. It has been reported that the exogenous expression of MTMR3 could suppress the growth of lung cancer cells^21^. In lung cancer cells, the increased expression of the *MTMR3* gene activates p27^21^. As reported, p27 is a cyclin-dependent kinase inhibitor that is mainly recognized as a negative regulator of the cell cycle. The overexpression of p27 triggers apoptosis in several different cancer cells^22^,^29^. Following the overexpression of p27, poly-(ADP-ribose) polymerase is cleaved and cyclin B1 is degraded, which is associated with apoptosis and G2/M phase cell cycle arrest. In our study, miR-100 silencing triggered the *MTMR3*-*p27* pathway and the activation of p27 induced the cleavage of poly-(ADP-ribose) polymerase, the accumulation of Bax and Bcl-2 and reduced cyclin B-CDK1 complexes, all of which are key proteins in apoptosis and G2/M cell cycle arrest. Silencing miR-100, therefore, induced cell cycle arrest and apoptosis of SK-BR-3 cells. This study revealed a novel miR-100-mediated pathway that prevented apoptosis of breast cancer cells. Considering the differential expression of miR-100 and the miR-100-mediated apoptotic pathways, the role of miR-100 in regulating apoptosis of breast cancer was different in various breast cancer cells.

Despite the high morbidity and mortality associated with breast cancer, there is currently little treatment for advanced breast cancer. DNA-damaging chemotherapy, radiation therapy and surgery are the main therapies of cancer. However, both chemotherapy and radiation have significant toxicities. Surgery has resulted in long-term survival of patients with early breast cancer, however, it often reoccurs years later in patients with advanced breast cancer. The results of our study suggest that miR-100 could be a promising target for the treatment of breast carcinoma, because miR-100 silencing could significantly prevent growth by inducing cell cycle arrest and apoptosis in breast cancer cells. In addition, our study showed that miR-100 antagonism could sensitize breast cancer cells to chemotherapy, suggesting that the efficacy of chemotherapy could be improved with less toxicity. Taken together, these findings reveal a potential target for the therapy of breast cancer.

## Materials and Methods

### Cell culture

Breast cancer cell lines MCF7, T47D, HCC1954, MDA-MB-453 and SK-BR-3 were purchased from ATCC and SUM149 was purchased from Asterland. MCF7, T47D, HCC1954 and SK-BR-3 were cultured in RPMI medium 1640 (Gibco, USA) with 10% FBS (Gibco, USA). MDA-MB-453 was cultured in Leibovitz’s L-15 medium (Sigma, USA) supplemented with 10% FBS. SUM149 was cultured in Ham’s F-12 medium (Invitrogen, USA) supplemented with 5% FBS, 5 μg/mL of insulin (Beyotime, China) and 1 ug/mL of hydrocortisone (Sigma, USA). MCF7, T47D, HCC1954, SUM149 and SK-BR-3 cells were cultured at 37 °C in a humidified atmosphere with 5% CO2 and MDA-MB-453 was cultured at 37 °C with 100% humidified atmosphere. The cell lines were profiled routinely by short tandem repeat analysis.

### Quantitative real-time PCR for miRNA detection

Total RNAs were extracted using a RNA isolation kit (Ambion, USA) according to the manufacturer’s instructions. Expressions of miRNAs were quantified using the standard TaqMan Micro-RNA assay (Applied Biosystems, USA). U6 (Applied Biosystems) was used as a control. Relative quantities of individual miRNA were calculated with the 2^−(ΔΔCt)^ method^8^.

### miRNA inhibitor and precursor transfections

The anti-miRNA-100 oligonucleotide (AMO) and the miR-100 precursor were transfected into cells with Lipofectamine RNAiMax transfection reagent (Life Technology, USA) at the final concentration of 50 nM according to the manufacturer’s protocol. The cells were cultured for 6–8 h and then the medium was replaced with fresh medium. The sequence of AMO-miR-100 was 5′-TT**C**GG**A**TC**T**A C*G*GG*T*T-3′, which was modified with locked nucleic acids (LNA; old letters), 2’-O-methyl (OME; Italic letters) and phosphorothioate (the remaining nucleotides). AMO-miR-100-scrambled (5′-GT**C**GG**T**TC**T**GA*T*GT*C*A-3′) was synthesized using the same modifications as above. The miRNAs were synthesized by Sangon Biotech (Shanghai, China). The miR-100 precursor and the negative control were purchased from Applied Biosystem (USA).

### Cell viability and proliferation analysis

Cell viability and proliferation analyses were conducted with MTS [3-(4, 5-dimethylthiazol-2-yl)-5-(3-carboxymethoxyphenyl)-2-(4-sulfophenyl)-2H-tetrazolium, inner salt] assays (Promega, USA). Cells were seeded onto a 96-well plate containing 100 μl of culture medium and 20 μl of MTS reagent was added. Subsequently, the plate was incubated for 2 h at 37 °C in a humidified incubator containing 5% CO_2_. The absorbance was recorded at 450 nm. Cell proliferation rate analysis was performed via calculating cell viability of time-course assays. All experiments were biologically repeated three times.

### Detection of caspase 3/7 activity

Caspase-Glo 3/7 assays (Promega) were used to detect the caspase 3/7 activity in cells according to the manufacturer’s protocol. Cells were seeded onto a 96-well plate before the medium was removed and 100 μl of Caspase-Glo 3/7 reagent was added. The mixture was incubated at room temperature for 2 h and the luminescence was analysed.

### Apoptosis detection with Annexin V

An apoptosis assay using a FITC Annexin V apoptosis detection kit I (Becton, Dickinson and Company, USA) was conducted according the manufacturer’s protocol. Cells were harvested and rinsed in cold phosphate-buffered saline (PBS), followed by resuspension in 1 × annexin binding buffer at 1 × 10^6^ cells/mL. 5 μl of Alexa Fluor488 Annexin V and 0.1 μg of PI (propidium iodide) were then added to the cells. Samples were incubated at room temperature for 15 min in the dark. 400 μl of 1 × annexin binding buffer was then added to the sample. The samples were analysed with a flow cytometer at an excitation of 575 nm.

### Tumourigenicity in nude mice

Experiments to evaluate the effects of miR-100 on solid tumours were performed as shown by the diagram chart. SK-BR-3 cells were collected at 5 × 10^6^ cells/ml in physiological saline. Matrigel (Becton, Dickinson and Company, USA) was added to the cell suspension at a ratio of 1:2. 100 μl of cell suspension was then subcutaneously injected into NOD/SCID mice to induce tumour growth. Ten d later, when the tumour volume was around 12.5 mm^3^, mice were injected via the lateral tail vein with 80 mg/kg of AMO-miR-100 or AMO-miR-100-scrambled every 3 days. The tumour volume was measured every 3 days. Forty-six days after tumour challenge the mice were sacrificed. The tumour sizes and tumour weights were examined. The expression levels of miR-100 in the solid tumours were measured by real-time PCR. Animal experiments were approved by The Animal Experiment Center of Zhejiang University, China. All the methods were carried out in “accordance” with the approved guidelines.

### Screening for miR-100 target gene with DNA microarray

To screen for the target genes of miR-100, Human Genome U133 Plus 2.0 Array (Affymetrix California, USA) was used. The Gene Expression Omnibus accession number is GSE53909. The SK-BR-3 cells were transfected with AMO-miR-100 or AMO-miR-100-scrambled. RNAs were extracted from cells and subjected to the DNA microarray 48 h after transfection. Non-treated cells were used as controls. DNA microarray analysis was conducted by Shanghai Biotechnology Corporation (Shanghai, China). Dual-channel detection was performed to discover the differences in gene expression^30^. Data normalization was performed using the cyclic LOWESS (Locally-weighted Regression) method to eliminate system-related variations.

### Target gene prediction of miR-100

The targets of miR-100 were predicted using Targetscan^31^, miRanda^32^, Pictar^33^ and miRInspector^34^ algorithms. The seed sequence (the second to the seventh nucleotides) of a miRNA was complementary to the 3’ untranslated region (3’ UTR) of its target mRNA. Predictions were ranked based on the predicted efficacy of targeting as calculated using the context+ scores from the sites.

### Dual-luciferase reporter assay

A dual-luciferase reporter assay (Promega) was conducted to investigate the interaction between miR-100 and its predicted target *MTMR3* gene. The *MTMR3* 3′UTR (5′-CCAAGAGGUUAUGAUACGGGUU-3′) and *MTMR3* 3′UTR mutant (5′-CCAAGAGGUUAUGACCATTAGU-3′) were cloned into the pmirGLO dual-Luciferase vector (Promega) to generate the MTMR3 3′UTR and MTMR3 3′UTR-mutant constructs. In addition, miR-100 (5′-AAC CCGUAGAUCCGAACUUGUG-3′) was cloned into a miExpress vector (Genecopoeia, USA). The SK-BR-3 cells were co-transfected with miExpress-miR-100 (miR-100) or miExpress plasmid (vector only) and MTMR3 3′UTR or MTMR3 3′UTR-mutant. Forty-eight h later, the firefly luciferase and renilla luciferase activities were detected according to the manufacturer’s protocol.

### Quantification of mRNA with real-time PCR

Total RNAs were extracted using an RNA isolation kit (Ambion). The reverse transcription reaction was conducted using PrimeScript™ RT Reagent Kit (Takara, Japan) and qPCR was conducted with 2 × TaqMan Premix Ex Taq (Takara, Japan). *β-actin* was included for normalization. The 2^−(ΔΔCt)^ method was used to calculate the relative fold change of mRNA expression^8^. The genes quantified were *MTMR3* (primers, 5′-AGTGTCAAGAGTGGCTGAAGAG-3′ and 5′-ATAGACCTCCATGCA CCAAGC-3′; probe, 5′-FAM-TGAACAACGCAATCCGACCACCT–Eclipse-3′), *p27* (primers, 5′-TGTGTAGAAAGTGAAATCAGAGGAG-3′ and 5′-CAGGAGTGATAT TATCTGGGTAAGC-3′; probe, 5′-FAM–CGGACCTCG GACAGGTGATCCACC-Eclipse-3′) and *β-actin* (primers, 5′-GACTACCTCATGAA GATCCTCACC-3′ and 5′-TCTCCTTAATGTCACGCACGATT-3′; probe 5′-FAM-CGGCTACAGCTTCACCA CCACGGC-TAMRA-3′).

### Western blot

Cells were lysed with RIPA buffer (Beyotime, China) containing 2 mM of phenylmethanesulfonyl fluoride. The samples were then subjected to an SDS-PAGE. After electrophoresis for 45 min at 200 V, the proteins were transferred to a polyvinylidene fluoride (PVDF) membrane (Millipore, USA). The membrane was then blocked with blocking buffer (5% milk in Tris-buffered saline and Tween-20) for 1 h at room temperature. The membrane was incubated with a primary antibody at 4 °C overnight. After three washes with Tris-buffered saline the membrane was incubated with alkaline phosphatase-conjugated secondary antibody (Roche, Switzerland) for 2 h at room temperature. The membrane was detected with BCIP/NBT substrate (Sangon Biotech, Shanghai, China). The primary antibodies against cleaved caspase 3, poly ADP-ribose polymerase, cleaved poly ADP-ribose polymerase, cylcin B, p-cyclin B, CDK1, p-CDK1, Bax, Bcl-2, MTMR3, p27 and β-actin were purchased from Cell Signal Technology (USA).

### Flow cytometric analysis of the cell cycle

SK-BR-3 cells were harvested and washed with PBS and then fixed with 70% precooled ethanol at 4 °C overnight. The cells were collected by centrifugation at 500 × g for 5 min. The pellets were rinsed with PBS and resuspended in PBS containing DNase-free RNase A (20 μg/mL) and PI (50 μg/mL). After incubation for 30 min at room temperature, the cells were measured by flow cytometry at an excitation wavelength of 488 nm.

### Silencing, overexpression and rescue of *MTMR3* gene expression in breast cancer cells

To knock down the expression of *MTMR3* in SK-BR-3 cells, the MTMR3- specific siRNA (*MTMR3*-siRNA, 5′-CAAUACUAUGCCCAAAGAACCUU-3′) was synthesized (Genepharma, China). MTMR3-siRNA was complementary to the coding sequence of *MTMR3* open reading frame (ORF). The sequence of MTMR3-siRNA was scrambled to generate the control siRNA (random-siRNA) (5′-GAAUACAAACC CUCACCGAAUUU-3′). SK-BR-3 cells were transfected with siRNA (50 nM) using Lipofectamine RNAiMAX reagent (Life Technology) according to the manufacturer’s instructions. The cells were collected at 48 h after siRNA transfection.

To overexpress the *MTMR3* gene, the *MTMR3* ORF was cloned into pcDNA (3.1) (Life Technology) using *MTMR3*-specific primers 5′-CA*GGATCC*ATGGATG AAG AGACTCGGCACAGC-3′ (BamHI sites in italic) and 5′-TC*GAATTC*TCAGTTGGA AGTGGCAGCAATGGGC-3′ (EcoRI sites in italic). In this construct, the *MTMR3* gene was synonymously mutated at two nucleotides (position 307 C→T and position 310 T→C) to prevent recognition by MTMR3-siRNA. The plasmids were confirmed by sequencing. The plasmid was transfected into SK-BR-3 cells using Attractene transfection reagent (Qiagen, Germany). Thirty-six h later, the cells were harvested for subsequent assays.

To rescue *MTMR3* expression in MTMR3-silenced cells, the SK-BR-3 cells were transfected with MTMR3-siRNA to silence the expression of the endogenous *MTMR3* gene. Twenty-four hours later, the pcDNA-∆MTMR3 plasmid was transfected into the MTMR3-siRNA-treated cells to rescue the expression of MTMR3. Cells were collected for subsequent analysis 36 h after the last transfection.

### Chemotherapy treatment of miR-100 knockdown breast cancer cells

SK-BR-3 cells were seeded and cultured in RPMI 1640 medium until the cells were 50% confluent. The cells were then transfected with AMO-miR-100 or AMO-miR-100-scrambled at a final concentration of 50 mM. Six h later, the medium was replaced with fresh medium containing Cisplatin at different concentrations. After culture for 48 h, the cells were subjected to cell viability and apoptosis analyses.

### Statistical analysis

All data were presented as mean ± standard deviation. Statistical significance was analysed using a Student’s *t* test.

## Additional Information

**How to cite this article**: Gong, Y. *et al.* The role of miR-100 in regulating apoptosis of breast cancer cells. *Sci. Rep.* **5**, 11650; doi: 10.1038/srep11650 (2015).
